# Hyperventilation in Severe Traumatic Brain Injury Has Something Changed in the Last Decade or Uncertainty Continues? A Brief Review

**DOI:** 10.3389/fneur.2021.573237

**Published:** 2021-03-11

**Authors:** Daniel Agustín Godoy, Rafael Badenes, Chiara Robba, Francisco Murillo Cabezas

**Affiliations:** ^1^Neurointensive Care Unit, Sanatorio Pasteur, Catamarca, Argentina; ^2^Intensive Care Unit, Hospital San Juan Bautista, Catamarca, Argentina; ^3^Department Anesthesiology and Surgical-Trauma Intensive Care, Hospital Clinic Universitari de Valencia, Valencia, Spain; ^4^Department of Surgery, University of Valencia, Valencia, Spain; ^5^INCLIVA Research Medical Institute, Valencia, Spain; ^6^Department of Anaesthesia and Intensive Care, Policlinico San Martino Istituto di Ricovero e Cura a Carattere Scientifico (IRCCS) for Oncology and Neuroscience, Genoa, Italy; ^7^School of Medicine and Surgery, University of Milano - Bicocca, Monza, Italy; ^8^Critical Care Medicine, Intensive Care Unit, Virgen del Rocío University Hospital, Sevilla, Spain

**Keywords:** hyperventilation, intracranial hypertension, intracranial pressure, hypocapnia, cerebral ischemia, cerebral hypoxia, severe traumatic brain injury

**Graphical Abstract:**
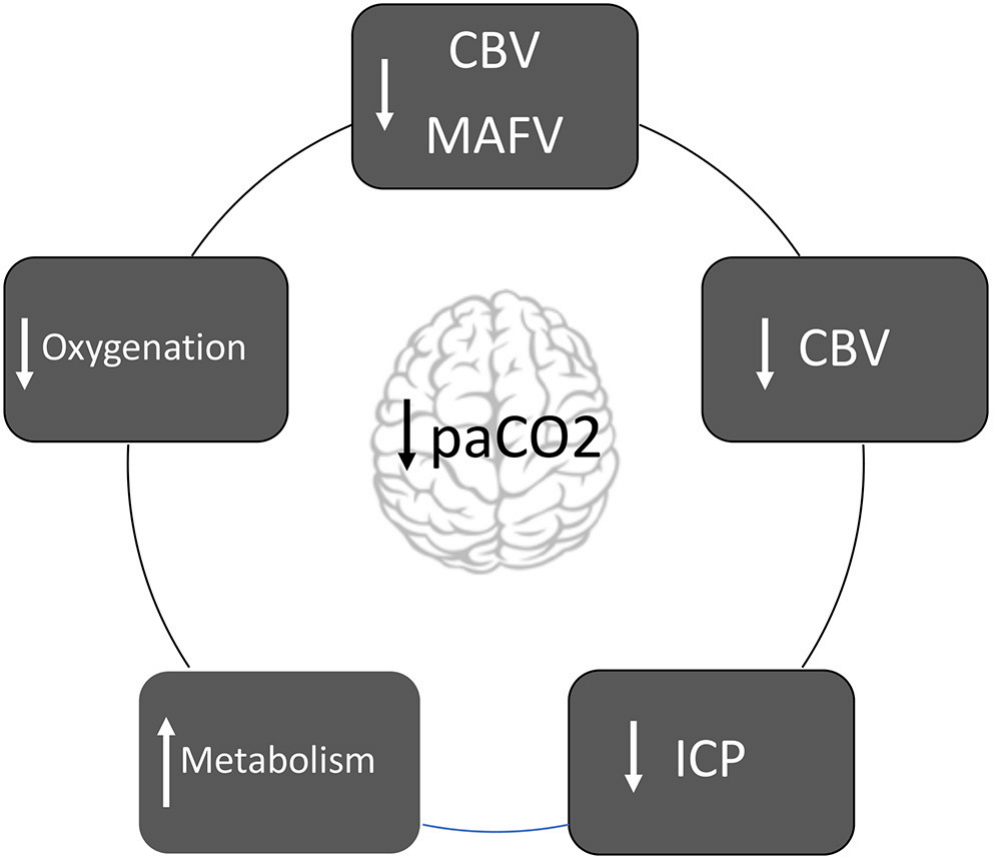
Diagram about the mechanisms in which hyperventilation affects the brain pathophysiology.

## Introduction

Induced hypocapnia through hyperventilation is a second-line measure to control intracranial pressure (ICP) when it remains elevated despite first line therapies (1). However, is not exempt of severe complications. Current recommendations of the Brain Trauma Foundation based on a level IIB evidence suggested against the use of hyperventilation up to a profound level (paCO_2_ < 25 mmHg), nor prophylactically and neither for a long period of time (2). Additionally, hyperventilation should be avoided during the first days after trauma when cerebral blood flow (CBF) is critically reduced (2). Lastly, when hyperventilation is necessary, a brain oxygen monitoring is mandatory (2). Focal brain oxygen monitoring is not the only adjunctive monitoring technique that can provide reassurance of the safety of hypoventilation. Jugulovenous oxygen saturation monitoring, for instance, is a much less expensive intervention with a fairly strong evidenciary base (2).

Since these recommendations are based on a low level of evidence, it is clear that certain controversies persist. A literature search of PubMed, Medline, Current Controlled Trials, and EMBASE was performed. The following search terms were used: hyperventilation and severe traumatic brain injury. Details of the studies were recorded using a dedicated data-extraction form. Titles, abstracts, or both, of studies retrieved using the search strategy and those from additional sources were screened independently, and the full text of potentially eligible studies was retrieved and assessed independently for eligibility. Disagreement over eligibility was resolved through open discussion.

Some questions are not yet answered with certainty and the results of recent studies motivate the following points of view:

## What Happens to the Brain Physiology During Hyperventilation?

Cerebral blood vessels (< 50 μm) are able to change their diameter when paCO_2_ levels change through the phenomenon called “CO2 reactivity” (3). Dilatation occurs with hypercapnia (paCO_2_ >44 mmHg), and constriction occurs with hypocapnia (paCO_2_ <35 mmHg) (3). This vascular activity occurs when paCO_2_ levels are between 20 and 60 mmHg, although the upper limit is not well defined. The response of CBF to changes in paCO_2_ resembles a sigmoid curve (3).

Changes in vessel diameter elicited by hypocapnia and hypercapnia are not proportional (4, 5). If paCO_2_ increases above 80 mmHg, vasodilation increases CBF around 100–200%, triggering catecholamines release and increase of metabolism. During hypocapnia, for every mmHg of paCO_2_ decrease, CBF decreases by 3%; thus, paCO_2_ levels between 20 and 25 mmHg are associated with a CBF reduction of 40–50% (4, 5).

Regarding vascular reactivity, the endothelium reacts to changes in perivascular pH by releasing mediators that regulate the state of smooth muscles (6). These vasoactive mediators include nitric oxide, prostaglandins, cyclic nucleotides, potassium, and calcium (6).

CBF is heterogeneous and changes according to the metabolic activity of each cerebral region (7). In fact, CO_2_ reactivity is not uniform (7). During the early phases of severe TBI, CO_2_ reactivity is exacerbated, especially in the areas adjacent to contusions or subdural hematomas. For these reasons, changes in normal levels of CO_2_ are potentially dangerous secondary insults that can drastically impact on brain physiology.

Cerebral blood volume (CBV) is 3–4 ml per 100 grams of cerebral parenchymal. Seventy percent of the total blood volume is contained in the venous system that does not react to changes in PaCO_2_ (7). Modifications in CBV are attributed only to blood contained in the arterial system (30%). For each mmHg of paCO_2_ reduction, CBV decreases by approximately CBV decreases by ~0.049 ml/100 grams of parenchyma. Therefore, if hypocapnia induces 30% of CBF decrease, CBV decreases only 7% (7). In summary, decrease in the paCO_2_ decreases CBF, but has little effect on CBV and ICP7. Finally, CBV response to hypocapnia is exacerbated when arterial hypotension is present (7).

Following the Monro-Kellie doctrine, if hypocapnia induces vasoconstriction and CBV decrease, ICP decrease consequently (8). Hypercapnia triggers vasodilation, which leads to an increase in CBV and a subsequent increase in ICP (8).

The ability of cerebral vessels to modify their diameter to keep a constant CBF across a range of perfusion pressures, called “cerebral autoregulation,” is a natural survival mechanism that works within certain limits. Preliminary studies with the evaluation of indices obtained from transcranial doppler (Mx, Prx) have shown that cerebral autoregulation improves with hyperventilation; however, this relationship is transient and works only with moderate levels of hypocapnia (8–11).

Hypocapnia induces the release of excitatory amino acids (*N*-Methyl-D-aspartate and glutamate) and increases both glucose consumption, and metabolic rate of O_2_ (CMRO2) (1). It also potentiates neuronal excitability and prolongs convulsive activity (1).

## Why Is Hyperventilation Potentially Dangerous?

Hyperventilation-induced hypocapnia is a potent stimulus, which causes vasoconstriction, therefore decreasing CBF and can potentially cause cerebral ischemia (1, 8).

Tissue hypoxia is defined when the amount of oxygen supplied to the cells is insufficient or when the cells—despite an adequate supply—are not able to metabolize it (8). Several clinical studies have showed that hyperventilation significantly reduces CBF and oxygen delivery (12–14).

Positron emission tomography (PET) was applied in some studies (15, 16) in patients without intracranial hypertension, decreasing paCO_2_ levels from 36 to 29 mmHg. Results from these studies demonstrated that hyperventilation decreased CBF and increased the number and volume of hypoperfused areas. However, these changes were not associated with changes in global (SjvO2, AVDO2) or local (pbtO2) oxygenation reduction. Zones in the range of hypoperfusion showed less reserve capacity to extract oxygen, which increased the risk of ischemic damage (15, 16).

In another study, the 28% of hyperventilated individuals showed a marked decrease in cerebral metabolic rate of oxygen (CMRO2) (17).

Similarly, Marion et al. (18) analyzed regional CBF and tissue hypoxia markers before and after hyperventilation at a target of 24.6 mmHg in individuals without intracranial hypertension. “Apparently healthy areas” adjacent to contusions or subdural hematomas were analyzed at 24–36 h and 3–4 days post-trauma. After hyperventilation, CBF decreased and an increase in glutamate, lactate, and lactate/pyruvate relationship was observed (18). The author's conclusion was that in brain parenchyma adjacent to analyzed areas, even brief periods of hyperventilation in the acute phase of trauma can significantly increase the risk of secondary brain injury (18).

Diringer et al. (19, 20) tested PET variables after hyperventilation (paCO_2_ 30 mmHg) in patients with and without ICP increase. CBV, CBF, and cerebral venous oxygen were decreased; however, there was no ischemia or energy dysfunction since CMRO2 remained unchanged at the expenses of oxygen extraction fraction (OEF) increase (19, 20) (Table 1).

**Table 1:** Effect of hyperventilation on cerebral blood flow and metabolic parameters.

**Author**	**Method**	**Moment of study post** **Trauma (n/time)**	**ICP** **(mmHg)**	**paCO_**2**_ target (mmHg)**	**Findings**
Cold et al. (5)	Xe-CT	15/2 days 16/3–7 days 8/2 week 6/3 week	19	26	Decrease rCBF
Coles et al. (15)	PET	4/24 h	19.5	29	Decrease ICP/CBF
		21/2–4 days 8/5–7 days			Increase CPP SvJO2/AVO2 without changes
Menon et al. (16)	PET	37–160 h	15	29	Decrease CBF/pvO2 Increase OEF CMRO2/CBV without changes
Coles et al. (17)	PET	15–240 h	17	29	Decrease CBF Increase OEF/CMRO2/ischemic brain volume 28% CMRO2 decrease
Marion et al. (18)	Thermodiffusion Microdialysis	24–36 h 3–4 days	16 19.6	26.1 24.9	Decrease CBF Increase glutamate, lactate, L/P
Diringer et al. (19)	PET	11.3 (8–14) h	14 (6–26)	30	Decrease
					CBF/CBV/CvO2
					Increase OEF
					CMRO2 without
					changes
Diringer et al. (20)	PET	11.3 (8–14) h1–5 days	14 24	30 25	Decrease CBF/CBV/CvO2 Increase OEF CMRO2 without changes

*Xe-CT, xenon-computed tomography; PET, positron emission tomography; ICP, basal intracranial pressure; rCBF, regional cerebral blood flow; CBF, cerebral blood flow; CPP, cerebral perfusion pressure; SvJO_2_, venous saturation of jugular bulb; AVO_2_, oxygen arteriovenous difference; pvO_2_, oxygen venous pressure; OEF, oxygen extraction fraction; CMRO_2_, cerebral metabolic rate of oxygen; CBV, cerebral blood volume; L/P, lactate-piruvate relationship; CvO_2_, venous content of oxygen*.

Hyperventilation-induced hypocapnia is one of the avoidable cerebral secondary insults. Multiple clinical studies have shown the direct relationship between low levels of paCO_2_ and decrease in global, such as saturation in the jugular bulb (SvjO2) or local parameters, such as tissue oxygen pressure (ptiO2) (21–25).

Hyperventilation can cause hypoxia through various mechanisms: (a) decrease CBF; (b) compromise of pulmonary ventilation-perfusion relationship; (c) deviation to the left of the oxygen-hemoglobin dissociation curve and (d) increased metabolic demands (1, 8). This phenomenon is not limited to brain vessels. The myocardial, intestinal, or renal vasculature are also affected; therefore, hyperventilation also has a negative systemic impact (1, 8).

## Is There a Solid Evidence That Associates the Hyperventilation With a Poor Outcome?

Only one prospective, controlled, and randomized study evaluated the association of hyperventilation with outcome (26). Three groups were analyzed: group 1: normoventilation (paCO_2_ 35 mmHg); group 2: hyperventilation (paCO_2_ 25 mmHg), and group 3 hyperventilation and THAM (tromethamine). Favorable outcome at 3 and 6 months from the event were significantly lower in the hyperventilation group; however, after 12 months, the differences between the groups were not significant. Of note, there was no evidence of ischemia in any of the three groups (26).

The conclusions of this study should be interpreted with caution. In fact, clinical and imaging characteristics were not well-balanced between the groups. Also, there was a small number of patients per group (type α error). The control group was hyperventilated (paCO_2_ 31 mmHg), and only 14% of the individuals in groups 1 and 2 respectively and 5% of group 3 had intracranial hypertension. Finally, when analyzing the final outcome at 12 months post-trauma, the best results correspond to hyperventilation + THAM group (26).

## Is There New Evidence About the Role of Hyperventilation in the Management of Severe Traumatic Brain Injury (tbi)?

Recently, two manuscripts were published regarding the use of hyperventilation in TBI (27, 28). In the first one, Wettervik et al. in a retrospective series concluded that hyperventilation in a mild range (paCO_2_: 30–34 mmHg) is safe and can improve cerebrovascular reactivity (27). Some considerations deserve to be expressed not to misunderstand the results. First, hyperventilation was induced in the absence of intracranial hypertension (mean ICP between 11 and 14 mmHg). Second, the study lacks monitoring of CBF, cerebral oxygenation, and neuroimaging follow-up that would allow to rule out the occurrence of ischemic complications; therefore, this study does not allow to demonstrate the safety of hyperventilation. Also, the analyzed population showed space-occupying lesions <25 cc with open basal cisterns (Marshall II) and does not specify the location of the microdialysis catheter, which is of extreme importance, since it only obtains data from an area no larger than 1.5 cm^2^ in a pathology characterized by dynamism and heterogeneity.

Additionally, prophylactically hyperventilated patients fluctuated during the first 3 days between making up 25 and 33% of the total of the analyzed population, while the mean paCO_2_ levels of all patients remained in the normoventilation range (35–37 mmHg) throughout the period of study (27). Although the energy metabolism is not modified, this can be explained in different ways. First, the absence of intracranial hypertension.

Second, the position of the microdialysis catheter, which perhaps was implanted in a healthy area without major metabolic compromise. Third, the population that was studied did not specifically direct to those truly hyperventilated (25–33% of total). Of note, the relationship between paCO_2_ levels and cerebral autoregulation is interesting, although the data should be interpreted with caution in the context of the above-mentioned limitations (27).

In the another study, Brandi et al. concluded that moderate hyperventilation (paCO_2_: 30–35 mmHg), for a short duration period, does not induce alterations in metabolism or cerebral oxygenation in a cohort of severe TBI (28). The study reaffirms previous pathophysiological concepts about hyperventilation; however, certain reflections are important. First, moderate hyperventilation was utilized in absence of ICP increase (average ICP 16 mmHg) (28). Second, probes were placed in the white matter of the most damaged cerebral hemisphere, in regions of normal appearance on the CT scan. We agree with the decision regarding the monitoring site, but as the authors signaled, PbrO2 only measures local interstitial oxygen availability of a very small area (28). Severe TBI is a heterogeneous condition, and not all areas respond in the same way to HYPERVENTILATION (9). Ischemic volume increases during hyperventilation, inclusive without detection of cerebral oxygen monitoring (17, 18).

Third, patients were included on average at 23 hours after trauma (28).

Fourth, there are some considerations about ventilatory management that need to be mentioned: (a) Due to the risk of alveolar distention, increased intrathoracic pressure, and decreased cerebral venous return with consequent ICP increase, hyperventilation with tidal volume increase is not recommended (1, 2, 8). (b) On the other hand, the patients were in supranormal pa02 values (147 mmHg), which contributes to masking possible declines in PbrO2 (29). In this context, Dellazio et al. described the usefulness of the pbtO2/paO2 relationship to detect episodes of hidden hypoxia, defined by a ratio below 0.10 (29). In the analyzed Brandi's cohort, the PbtO2/paO2 ratio was 0.20, close to the mentioned value.

Fifth, mean arterial blood pressure (MABP) and cerebral perfusion pressure (CPP) were 92 and 77 mmHg, respectively. Mean CBF velocity of both mean cerebral arteries was 80 cm/s, while the mean PbrO2 value was 32 mmHg (27). In our opinion, these values are unusually elevated for patient's requirement, so, this could explain why moderate hyperventilation did not induce ischemic changes.

## Do the Results of the Recent Studies Justify Changing Our Usual Clinical Practice?

According to the available evidence, our point of view is not to change the current practice of avoiding hyperventilation in the context of severe TBI in the absence of ICP elevation. All efforts should be directed to avoid hypocapnia especially the first 24 h of trauma (30).

## Are there Circumstances That Allow the Use of Hyperventilation?

Hyperventilation in the setting of imminent increases in ICP is not explored fully—should we really even be doing that? This is the real question because it is common practice to avoid hyperventilation otherwise.

We cannot afford to easily discard anything useful of HYPERVENTILATION (1, 2). By contrast, we believe that hyperventilation could be used for a short period of time with brain oxygenation monitoring when ICP is not controlled with first line therapies and in certain emergency situations (herniation syndromes, plateau waves, intracranial hypertension associated to hyperemia) as a “bridge” pending definitive solution (1, 2).

## Author Contributions

DG, RB, CR, and FM drafting the article, critical revision of the article, and final approval of the version to be published.

## Conflict of Interest

The authors declare that the research was conducted in the absence of any commercial or financial relationships that could be construed as a potential conflict of interest.
